# Annual noise exposure among Malaysian young adults

**DOI:** 10.1097/EE9.0000000000000511

**Published:** 2026-08-10

**Authors:** Kun Ling Liew, Nashrah Maamor, Wan Syafira Ishak, Shin Ying Chu, Onn Wah Lee

**Affiliations:** a Centre for Rehabilitation and Special Needs Studies (iCaRehab), Faculty of Health Sciences, Universiti Kebangsaan Malaysia, Kuala Lumpur, Malaysia; b Centre for Healthy Ageing and Wellness (H-CARE), Faculty of Health Sciences, Universiti Kebangsaan Malaysia, Kuala Lumpur, Malaysia

**Keywords:** Annual noise exposure, Noise Exposure Questionnaire, Noise-induced hearing loss, Young adults

## Abstract

**Background:**

Prolonged exposure to both occupational and recreational noise can lead to noise-induced hearing loss. This study aimed to determine annual noise exposure (ANE) levels among Malaysian young adults using the Noise Exposure Questionnaire and to examine differences in ANE by gender, race, and medical background.

**Methods:**

A culturally modified Noise Exposure Questionnaire was validated and distributed to 205 healthy young adults (mean age = 22.9, SD = 3.0). ANE values were computed based on participants’ reported frequency and duration of exposure, combined with predetermined mid-intensity levels for each activity. Test-retest reliability was assessed in 20 randomly selected participants after a 3-month interval.

**Results:**

The median ANE was 71.4 dBA (95% confidence interval [CI] = 70.5, 72.1). A total of 16.5% (n = 34) of participants exceeded the National Institute for Occupational Safety and Health-recommended limit of 79.0 dBA. Subgroup analyses using bootstrap CIs suggested a possible difference in ANE between males (73.8 dBA, 95% CI = 71.7, 76.1) and females (70.5 dBA, 95% CI = 69.2, 71.3), with the wider interval in males reflecting the smaller male sample (n = 49). ANE estimates were comparable between racial groups (Chinese: 70.8 dBA, 95% CI = 69.7, 71.7; Malay: 71.9 dBA, 95% CI = 66.9, 77.5), though the considerably wider CI in the Malay subgroup (n = 25) limits the interpretability of this comparison. ANE was similarly comparable between participants with (70.9 dBA, 95% CI = 69.7, 71.9) and without (71.5 dBA, 95% CI = 69.0, 73.3) a medical background. Approximately 85% of participants reported never using hearing protection during motorized vehicle use, amplified events, and musical instrument playing. Test-retest reliability showed a moderate intraclass correlation coefficient (ICC3 = 0.60, 95% CI = 0.22, 0.82), though the wide CI warrants cautious interpretation.

**Conclusion:**

Recreational and daily lifestyle habits among young adults may contribute to elevated noise exposure, increasing the risk of noise-induced hearing loss. These findings underscore the importance of promoting hearing health awareness and safe listening practices among youth to mitigate preventable hearing loss.

**What this study adds**

This study addresses a critical gap in environmental epidemiology by providing the first validated tool for assessing cumulative annual noise exposure in Southeast Asian young adult populations. Unlike studies focusing on discrete noise-exposure activities, we quantified total noise exposure across multiple daily activities, revealing that recreational sources can contribute to an individual exceeding the recommended threshold. The finding that prevention is severely hindered by poor hearing protection adoption identifies a critical intervention point. These findings inform environmental health policy by demonstrating modifiable exposure pathways and highlighting the need for integrated noise control strategies in rapidly urbanizing middle-income countries with limited financial accessibility to hearing rehabilitation.

## Introduction

Noise-induced hearing loss (NIHL) remains a significant yet preventable public health challenge globally. The World Health Organization (WHO) estimates that approximately 5% of the world population suffers from disabling hearing loss, with unsafe listening practices placing over 1.1 billion young individuals at risk of permanent hearing damage.^1,2^ This condition carries substantial economic consequences, costing the global economy an estimated $750 billion annually.^3^

Traditional occupational noise exposure guidelines, such as the National Institute for Occupational Safety and Health (NIOSH) recommended exposure limit of 85 dBA with a 3 dB exchange rate, have primarily focused on workplace settings.^4^ However, emerging evidence highlights lifestyle-related and recreational noise exposure as increasingly important contributors to NIHL risk among young adults. Rapid urbanization has intensified environmental noise exposure, with cities worldwide documenting harmful noise levels that frequently exceed the US Environmental Protection Agency’s recommended 24-hour exposure limit of 70 dBA for the general public.^5,6^ This environmental noise pollution compounds individual risk, as people tend to increase personal listening device (PLD) volumes to overcome ambient noise, thereby elevating their cumulative noise exposure.^7,8^ Recognizing the growing burden of recreational noise, the WHO, in collaboration with the International Telecommunication Union, recommends that leisure noise should not exceed 80 dB for a maximum of 40 hours per week, a threshold that may be exceeded in common recreational activities.^1,9–11^

Recreational noise exposure has become particularly concerning among young populations. A UK survey of 10,401 adults found that 73.0% experienced recreational noise exposure in the past year, yet only 2.1% consistently used hearing protection.^12^ Despite the proven effectiveness of hearing protection devices in preventing NIHL, consistently low usage rates among young adults worldwide highlight the need to understand barriers to protective behavior adoption. High-risk activities included nightclub attendance, live music events, and PLD use.^13–16^ The U.S. Centers for Disease Control and Prevention reports that half of young people listen to audio at unsafe volume levels,^17^ with studies showing 30.0% of young adults using PLDs at maximum volume daily.^18^ Additionally, video gaming has emerged as a significant noise source, with sound levels reaching up to 91.2 dBA in shooting games.^19^

Despite these global findings, comprehensive data on annual cumulative noise exposure among young adults in Southeast Asia, particularly Malaysia, remain limited. To address this gap, we employed the Noise Exposure Questionnaire (NEQ), a validated tool that assesses cumulative noise exposure over 12 months across occupational and nonoccupational activities, expressed as annual noise exposure (ANE).^20^ In the original validation study of 114 young adults (ages 18–19), researchers found an average ANE of 75.0 dBA, with 32.0% meeting high-risk criteria for NIHL and significantly higher exposure in males (78.0 dBA) versus females (73.0 dBA).^20^ The NEQ has since been cross-culturally adapted and validated in other populations, including a Chinese validation study of 641 adults, which reported a mean ANE of 77.6 dBA (SD = 7.4).^21^ The variation in ANE values across these studies highlights differences in noise exposure patterns between populations, further underscoring the need for population-specific data in the Southeast Asian context, where such estimates remain limited.

Several factors may influence noise exposure patterns among young adults. Gender differences have been consistently reported, with males traditionally showing higher exposure due to occupational and recreational activities.^22–24^ For instance, males showed higher participation in the workplace as well as activities associated with elevated noise exposure, such as visiting clubs or discotheques, attending sporting events and concerts, and listening to music at levels above recommended safety thresholds. However, recent evidence suggests narrowing gender gaps as female participation in noise-related activities increases.^25^ Cultural and ethnic factors may also influence music preferences, listening habits, and leisure activities, though limited research exists on racial differences in ANE values within Asian populations. Finally, individuals with medical training may demonstrate greater awareness of NIHL risks, potentially influencing their protective behaviors.

The objectives of this study were to: (1) culturally modify and validate the NEQ for Malaysian young adults, (2) determine ANE levels and hearing protection use patterns among healthy Malaysian young adults aged 19–39 years, (3) compare ANE values across gender, race, and medical background, and (4) assess test-retest reliability of the modified questionnaire. These findings aim to inform targeted public health interventions and hearing conservation strategies for young adults in Malaysia and similar populations.

## Methods

### Study design

**Figure 1:**
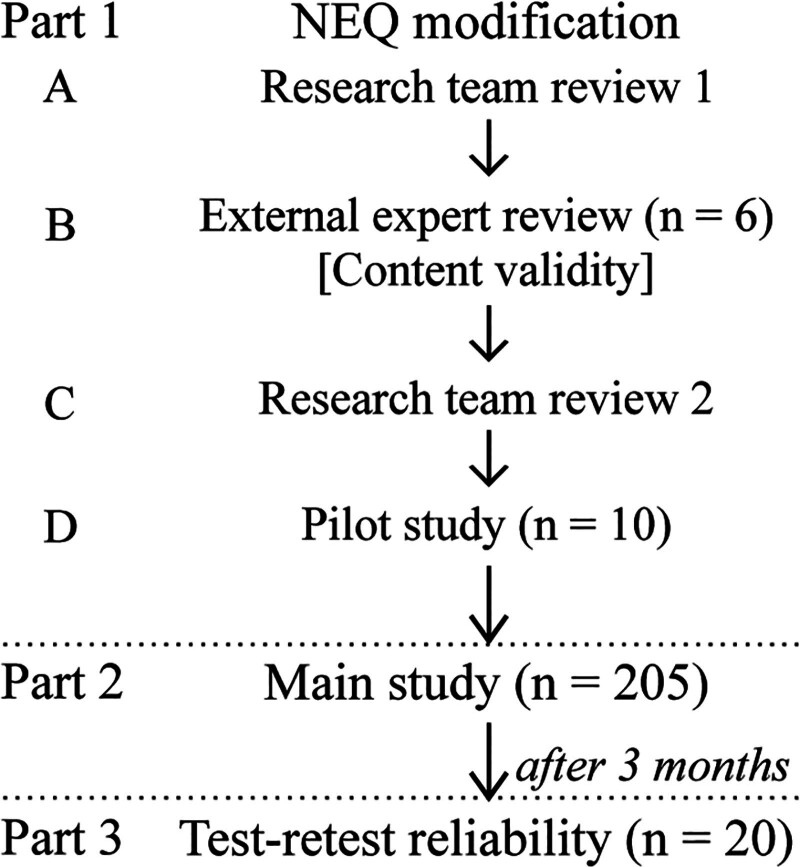
. Study flowchart.

This study consisted of three parts (Figure 1). Part 1 describes the modification of the NEQ to suit local culture. This section describes the procedures for establishing content validity, as well as a pilot study. Part 2 describes the average ANE values across 205 young adults, the comparison of ANE values between three factors: gender, race, and medical background, and reporting the hearing protection use patterns. Part 3 describes the test-retest reliability of ANE values. This study adhered to the Declaration of Helsinki, obtained approval from the Human Ethics Committee of the Universiti Kebangsaan Malaysia (JEP-2024-1126), and obtained informed consent from reviewers and participants.

### Part 1: instrument design—the Noise Exposure Questionnaire

Permission to use the NEQ was obtained from the original author, Tiffany A. Johnson.^20^ This questionnaire systematically assesses both occupational and nonoccupational noise exposure activities across four main domains: (1) occupational noise exposure (noisy paid employment), (2) housework activities (power tools, equipment/machinery use), (3) transportation (motorized vehicles, public transport), and (4) recreational activities (amplified events, musical instruments playing, and PLDs).

For each noise exposure activity, participants report two key components: (1) frequency of participation (ranging from “never” to “daily”) and duration of exposure per occasion (from “less than 1 hour” to “more than 8 hours”) and (2) frequency of hearing protection devices use during that activity (“never,” “sometimes,” or “always”). This dual approach enables simultaneous assessment of noise exposure risk and hearing protective behavior patterns.

In this study, hearing protection devices refer to earplugs or earmuffs. Consistent with the original NEQ framework, no attempt was made to adjust ANE estimates to account for the type or effectiveness of specific hearing protection devices, as the NEQ was not designed to evaluate the quality of individual protection devices.^20^

NEQ comprehensive approach provides advantages over traditional noise exposure assessments by capturing cumulative noise exposure across diverse activities and incorporating hearing protection usage patterns, making it particularly suitable for evaluating noise exposure in young adults whose primary risks may stem from recreational rather than occupational sources.

#### Part 1: participants

Part 1 involves two groups of participants. The first group consisted of six external experts (Figure 1, Part 1B), who were three academicians with at least 20 years of clinical experience and three audiologists with over 10 years of working experience. The second group consisted of 10 young adults (mean age = 23.3; SD = 1.2), who were recruited through convenience sampling (Figure 1, Part 1D). The inclusion criterion was young adults aged between 19 and 39 years, and the exclusion criterion was participants who were clinically diagnosed with hearing loss or had an ear infection.

#### Part 1: procedures

The original NEQ required cultural adaptation to ensure relevance for Malaysian young adults. The following questions were removed after the research team review (Figure 1, Part 1A): (1) How often were you around or did you shoot firearms such as rifles, pistols, and shotguns? (2) How often did you ride on or pilot small aircraft/private aircraft/private aeroplanes? (3) Over the summer months, did you work a noisy paid job, such as in construction, farming, a factory, lawn service, car wash, or other indoor or outdoor jobs, working around loud equipment or machinery? The justifications for these decisions were that the possession of a firearm is illegal in Malaysia, the uncommon activity of flying an aircraft due to airspace restrictions and tight regulations on a private pilot license, and no summer work opportunities like those overseas.

An additional item was added to the questionnaire (Question 10) addressing gaming with loud background sounds using speakers or PLDs. This addition reflects modern leisure trends among young adults, as video games can reach noise levels of up to 91.2 dBA.^26^ Additionally, we replaced culturally irrelevant examples (jet skis, speedboats, and snowmobiles) with locally relevant transport modes (monorail, Light Rail Transit).

The questionnaire was then sent for external expert review (Figure 1, Part 1B), including the three removed questions, to investigate their relevance. Each expert rated the relevance of each question using a Likert scale from 1 (not very relevant) to 5 (very relevant) and provided feedback or comments. The raw scores of 3 and below were given a rating of 0, which indicates not relevant, and scores of 4 and above were given a rating of 1, which indicates relevant. The content validity index for each item (I-CVI) was calculated by averaging the ratings across experts, and the overall content validity index (S-CVI) was calculated by averaging the I-CVI across items. The acceptable S-CVI value is at least 0.83 for a panel with six experts.^27^

Expert feedback regarding grammar and sentence structures was incorporated to improve questionnaire clarity (Figure 1, Part 1C), followed by a pilot study with 10 young adults (Figure 1, Part 1D). All pilot participants filled in the questionnaire, provided verbal feedback on unclear items, and confirmed that the questionnaire was clear and relevant to their experiences. The finalized questionnaire is attached as Appendix 1; https://links.lww.com/EE/A451.

#### Part 1: results

We computed the I-CVI for each item and the overall average, S-CVI, based on external expert ratings. As shown in Table 1, most items achieved the maximum I-CVI of 1.00, except for Item 3. The S-CVI was 0.98, higher than the recommended 0.83,^27^ suggesting the questionnaire is measuring what it is intended for. The three removed questions showed low relevance, with an I-CVI of 0.17, strongly indicating the need for their removal.

**Table 1.** The relevance rating on the items of the modified NEQ by six experts

Item	Description	E1^a^	E2		E3		E4		E5		E6	I-CVI^b^
Q1	Noisy job exposure	1	1		1		1		1		1	1
Q2	Power tool noise exposure	1	1		1		1		1		1	1
Q3	Heavy machinery noise exposure	1	0		1		1		1		1	0.83
Q4	Motorized vehicle noise exposure	1	1		1		1		1		1	1
Q5	Public transport noise exposure	1	1		1		1		1		1	1
Q6	Amplified events noise exposure	1	1		1		1		1		1	1
Q7	Musical instrument exposure	1	1		1		1		1		1	1
Q8	Personal listening device music exposure	1	1		1		1		1		1	1
Q9	Speaker music exposure	1	1		1		1		1		1	1
Q10	Gaming noise exposure	1	1		1		1		1		1	1
S-CVI^c^												0.98
Removed items												
R1	Summer work noise exposure	0		0		0		1		0	0	0.17
R2	Firearm exposure	0		0		0		1		0	0	0.17
R3	Aircraft noise exposure	0		0		0		1		0	0	0.17

a E denotes Expert.

b I-CVI = item-level Content Validity Index, which assesses the validity of each item being asked in the questionnaire.

c S-CVI = scale-level Content Validity Index, calculated based on the average I-CVI values.

### Part 2: instrument implementation: annual noise exposure of Malaysian young adults

#### Part 2: participants

This study recruited 211 young adults aged between 19 and 39 years using convenience and snowball sampling methods. The exclusion criteria were participants who were clinically diagnosed with hearing loss or had an ear infection, as these conditions could affect baseline noise exposure behaviors and hearing protection awareness. Only 205 healthy young adults (mean age = 22.9; SD = 3.0) were included in the final analysis. Three participants were clinically diagnosed with hearing loss, two had ear infections, and one was from a Sino-native (indigenous mixed Chinese and native Borneo heritage) background, who could not be categorized within the racial classification system of this study. Table 2 shows the demographic data of the participants.

**Table 2.** Demographic data (n = 205)

Factor	Subgroup	n	Age mean (SD^a^)
Gender	Male	49	22.3 ± 3.3
	Female	156	23.1 ± 3.0
Race	Chinese	180	22.8 ± 3.1
	Malay	25	23.8 ± 2.4
Medical background	Yes	145	22.5 ± 2.2
	No	60	23.9 ± 4.3

a SD denotes one standard deviation.

#### Part 2: procedures

The finalized questionnaire was distributed through Google Forms using social media and email, with no participation incentives offered. The data collection process was conducted over approximately three months.

#### Part 2: data analysis

##### Annual noise exposure calculation

ANE values were calculated following established NEQ protocols.^20,21^ Participants’ reported frequency and duration for each activity were converted to annual hours using predetermined conversion factors: frequency values (“never” = 0, “every few months” = 1, “monthly” = 12, “weekly” = 50, “daily” = 200) and duration values (“less than 1 hour” =1, “1–4 hours” = 3, “4–8 hours” = 6, “more than 8 hours” = 8), except for occupational noise exposure which was calculated directly from reported weeks and hours per week.

Each activity’s noise exposure dose was computed by integrating ANE hours with a representative sound level assigned to each activity (refer Table 1 in Johnson et al^20^). These representative sound levels, referred to as “mid” values, were pre-established by Johnson et al^20^ through a systematic review of published acoustic measurement literature. For activities originally queried by Neitzel et al^11^, the sound levels from that earlier work were adopted directly. For activities added in the NEQ, Johnson et al^20^ independently surveyed the relevant literature, including only studies that reported A-weighted sound levels measured at appropriate distances using representative rather than maximum levels. The mid-value, 88 dBA, assigned to gaming activity was derived from studies reporting measured A-weighted sound levels across various gaming setups, including both personal headsets and speakers.^19,28^

The ANE value is calculated as:


$$\begin{array}{c} ANE=\left[10\times \log_{10}\left(total\;dose/100\right)\right]+79 \end{array}$$

where total dose is the sum of individual activity doses across all 10 items. An ANE value ≥79.0 dBA was considered high-risk for developing NIHL, based on NIOSH criteria extrapolated from the occupational recommended exposure limit of 85 $L_{Aeq2000h}$ to an annual equivalent of 78.6 $L_{Aeq8760h}$.^20^ The final ANE value represents the equivalent continuous 24-hour noise exposure over an entire year (L_Aeq8760h_).

### Statistical analysis

To investigate ANE differences between (1) gender, (2) race, and (3) medical background, we calculated the median ANE and 95% confidence interval (CI) for each subgroup. Estimates were derived using bootstrap resampling (percentile method, 1000 iterations). This analysis was completed using the default boot function in RStudio 2026.04.0 + 526.

#### Part 2: results

##### Annual noise exposure levels and participation rates

The mean ANE value was 71.8 dBA (SD = 7.1). Notably, 16.5% (n = 34) of participants exceeded the NIOSH-recommended limit of 79.0 dBA. When stratified by gender (males = 49; females = 156), the proportions were comparable: 16.3% (n = 8) of males and 16.7% (n = 26) of females exceeded this threshold.

**Figure 2.**
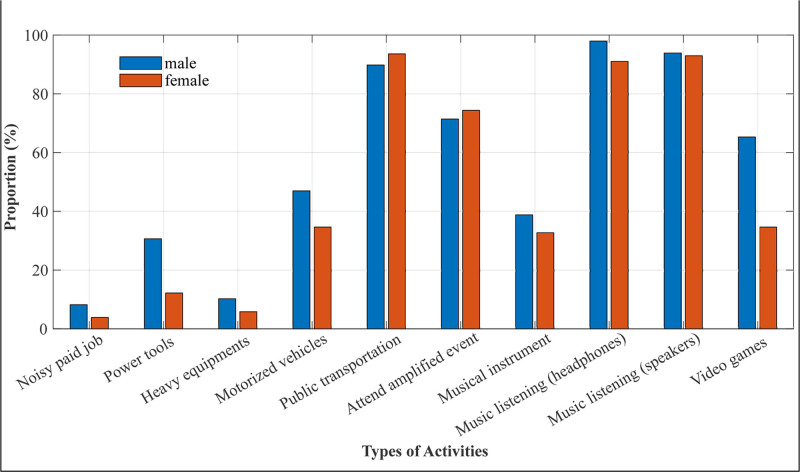
Participation in activity between males and females. Blue indicates male and red indicates female.

The five most common noise exposure activities were listening to music and radio programs via speakers (n = 191; 93.2%), listening to music and radio programs using personal headsets (n = 190; 92.7%), taking public transport (n = 190; 92.7%), attending events with amplified public announcements or music (n = 151; 73.7%), and playing video games using speakers or PLDs (n = 86; 42.0%). Males showed a higher participation rate in almost all activities except public transport use and attendance at amplified events (Figure 2), with proportions calculated within each gender group.

##### Comparison of annual noise exposure values across gender, race, and medical or nonmedical background

**Figure 3.**
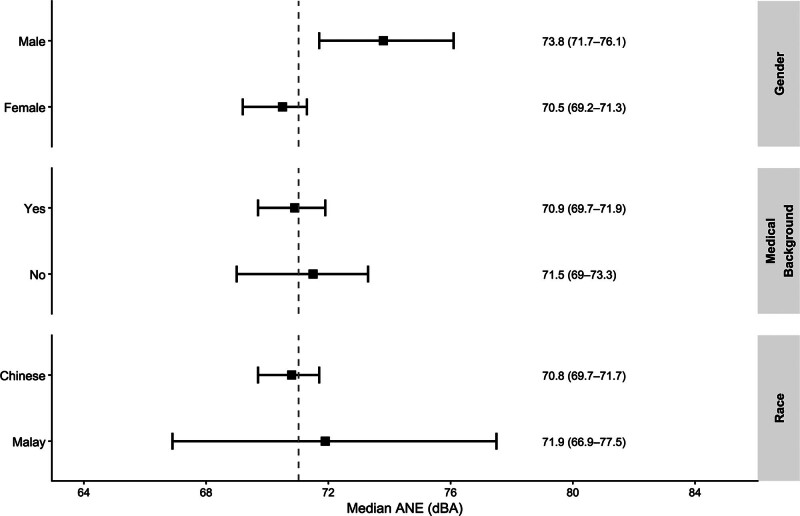
Median ANE in dBA with 95% confidence intervals by subgroup. Each square represents the median ANE for the subgroup, with square size proportional to sample size. Horizontal lines indicate the 95% confidence interval. The dashed vertical line represents the overall median ANE. A wider confidence interval indicates greater uncertainty in the estimate, largely attributable to smaller subgroup sample sizes.

Figure 3 shows the median ANE in dBA with 95% CIs by subgroup. Median ANE was slightly higher in males (73.8 dBA, 95% CI = 71.7, 76.2) compared to females (70.5 dBA, 95% CI = 69.2, 71.4). The CI for males was notably wider (width: 4.5 dBA) than for females (width: 2.2 dBA), reflecting the smaller male sample (n = 49) and greater uncertainty in that estimate. There was no overlap between the two intervals, suggesting a possible difference between sexes, though the difference was small and the precision of the male estimate limits firm conclusions.

Among racial groups, the median ANE was similar between Chinese (70.8 dBA, 95% CI = 69.7, 71.7) and Malay participants (71.9 dBA, 95% CI = 66.9, 77.5). However, the considerably wider CI for the Malay subgroup (CI width: 10.6 dBA) compared to Chinese participants (CI width: 2.0 dBA) reflects the substantially smaller Malay sample (n = 25) and limits any meaningful comparison between these groups.

Median ANE was comparable between participants with (70.9 dBA, 95% CI = 69.7, 71.9) and without (71.5 dBA, 95% CI = 69.0, 73.3) a medical background. The nonoverlapping CIs and small absolute difference of 0.6 dBA suggest little meaningful difference between these groups, though the wider interval in the “No” group (CI width: 4.3 dBA) indicates relatively greater uncertainty in that estimate.

##### Hearing protection use pattern

Participants who reported exposure to noise activities (Items Q1-Q7) were asked to rate their frequency of hearing protection use as *“*never,” “sometimes” or “always.” Items Q8–Q10 were not included in the assessment of hearing protection use, as these items pertain to activities that typically adopt noise reduction strategies other than hearing protection devices. Specifically, Q8 refers to listening to music via a PLD, Q9 refers to listening to music through speakers, and Q10 refers to gaming using a PLD or speakers, activities for which lowering the volume is the more applicable protective strategy.

Table 3 shows that approximately 85% reported never using hearing protection during three specific activities: motorised vehicles (Q4), attendance at events with amplified public announcements or music (Q6), and musical instrument playing (Q7). For remaining noise exposure activities, at least 40% of exposed participants indicated they never use hearing protection. Notably, fewer than 10% of participants in each exposed subgroup reported always using hearing protection across all activities.

**Table 3.** Frequency of hearing protection use by noise exposure activity (n = 205)

Item	Activity	Exposed, n (%)	Never, n (%)	Sometimes, n (%)	Always, n (%)
Q1	Noisy paid job	10 (4.9)	4 (40.0)	6 (60.0)	0 (0)
Q2	Power tools	34 (16.6)	18 (52.9)	14 (41.2)	2 (5.9)
Q3	Equipment/machinery	14 (6.8)	8 (57.1)	5 (35.7)	1 (7.1)
Q4	Motorized vehicles	77 (37.6)	66 (85.7)	11 (14.3)	0 (0)
Q5	Public transport	190 (92.7)	134 (70.5)	45 (23.7)	11 (5.8)
Q6	Amplified events	151 (73.7)	129 (85.4)	21 (13.9)	1 (0.7)
Q7	Musical instruments	70 (34.2)	60 (85.7)	7 (10.0)	3 (4.3)

Note: Percentages of hearing protection use frequency (never/sometimes/always) are calculated from participants exposed to each specific activity, not the total sample size.

### Part 3: instrument reliability: test-retest reliability study

#### Part 3: participants

Test-retest participants were recruited from the main sample of 205 participants. At the end of the initial questionnaire, all participants were invited to provide their contact information if they were willing to complete the same questionnaire again after 3 months for test-retest reliability assessment. Only 20 participants (9.8%) volunteered their contact information and agreed to participate in Part 3.

#### Part 3: data analysis

Test-retest reliability was assessed using intraclass correlation coefficients (ICC) with a two-way mixed-effects model with absolute agreement, reported as ICC3 with a 95% CI. Reliability was interpreted according to the benchmarks proposed by Koo and Mae (2016),^29^ where values below 0.50 indicate poor reliability, 0.50–0.75 moderate reliability, 0.75–0.90 good reliability, and above 0.90 excellent reliability. All reliability analyses were conducted using the psych package in RStudio 2026.04.0 + 526.

#### Part 3: results

The computed ICC3 was 0.60 (95% CI = 0.22, 0.82), indicating moderate reliability.^29^ However, the wide CI reflects considerable uncertainty in the estimate, attributable to the limited sample size available for the reliability analysis. A post-hoc brief interview was conducted with two participants, who had the highest disparity between the main and test-retest studies, about their thoughts when completing the test-retest study. Quotes of their responses are as follows:

Participant A: “When I first filled it out, I didn’t expect that I would end up going to the gatherings every week. But the gatherings weren’t particularly noisy—it was mainly piano music and everyone singing hymns together. That’s why I was a bit conflicted, and the answers I gave ended up being different. Also, it could be that the timing of when I filled it out was different, which affected the amount of time I reported participating.”

Participant B: “During that period, I was going back to my hometown more frequently, which increased my exposure to noise. I often listened to music using the car audio while driving back and forth. I also used the motorcycle more often when I was home as it is more convenient. On top of that, I had the habit of frequently using earphones to listen to music during that period of time.”

## Discussion

### Content validity of modified Noise Exposure Questionnaire

The modified NEQ demonstrated a high content validity index (CVI = 0.98), consistent with the Chinese validation study by Han et al,^21^ which reported a similarly high CVI score (CVI > 0.80 for all items), suggesting that the items included in the questionnaire are appropriate for assessing noise exposure across activities commonly experienced by young adults. Beyond content validity, the NEQ has demonstrated its utility in identifying individuals at risk of NIHL across diverse populations, including hunters,^30^ musicians,^31^ and young adults with high nonoccupational noise exposure.^32^ Collectively, these findings support the cross-cultural applicability of the NEQ and its relevance for use among Malaysian young adults.

### Prevalence of young adults at risk of noise-induced hearing loss

Our study found that 16.5% of young adults (age range: 19–39 years) in this cohort exceeded the recommended ANE limit of 79.0 dBA. This value is lower than the 32% reported in the original NEQ study conducted among college-aged participants (18–19 years old).^20^ The observed discrepancy is likely attributable to fundamental differences in the study populations rather than age per se. Johnson et al^20^ recruited college students, who may be characterized by a distinctly different lifestyle, including higher participation in social recreational activities, nightlife, and communal listening environments, compared with the broader young adult population in the current study, which encompasses both university students and working adults. These lifestyle differences, rather than age alone, may more meaningfully account for the observed discrepancy. Notably, the original study reported a substantially higher percentage of males exceeding safe noise levels (55.0% vs. 16.3% in this study), whereas female participants showed more comparable results (14.0% vs. 16.7% in this study), further suggesting that population characteristics and recreational behavior patterns may underlie these differences.

Despite these differences, the 16.5% prevalence remains concerning. This is particularly alarming in Malaysia, where hearing healthcare resources are less developed than in high-income countries. Malaysia’s health expenditure in 2022 was 2.9% of GDP according to World Bank data, below the 5.0% recommended by the Health Financing Strategy for the Asia Pacific Region (2010–2015). The financial burden of hearing loss management, including hearing aids, cochlear implants, and rehabilitation services, is exacerbated by limited insurance coverage and high out-of-pocket costs.^33,34^ This resource scarcity underscores the critical importance of prevention, as Malaysian young adults may face significant barriers to accessing appropriate hearing rehabilitation if NIHL develops.

Unsurprisingly, listening to music through headphones or speakers was the most frequently reported activity, which is consistent with previous research.^20^ Breinbauer et al.^35^ demonstrated that preferred listening levels often exceed safe thresholds, particularly in noisy environments. Their study found that 17.3% of the participants spontaneously chose volumes above 85 dB in quiet conditions. Alarmingly, in 90 dB noise environments, 74.8% of subjects preferred volumes exceeding 95 dB. These findings suggest that common activities, such as listening to music while walking on noisy streets or using public transportation (the third most reported activity in our study), may unconsciously prompt users to increase volumes to hazardous levels due to environmental noise masking, potentially causing hearing damage.

Our modified NEQ also revealed gaming as another prevalent activity, whereas items such as power tool use and operating heavy vehicles, which showed high participation in the original NEQ study, were less common in our cohort. These differences underscore the importance of culturally adapting NEQs to reflect local lifestyle patterns. Therefore, public health initiatives targeting NIHL should prioritize locally relevant behaviors, particularly unsafe music listening practices and gaming habits, to maximize their preventive impact.

### Comparison of annual noise exposure scores across gender, race, and medical or nonmedical background

The findings of this study suggest a possible difference in ANE between males and females, with males recording a higher median ANE (73.8 dBA, 95% CI = 71.7, 76.1) compared with females (70.5 dBA, 95% CI = 69.2, 71.3). The CI showed no overlap, suggesting a plausible difference; however, the wider CI observed in the male subgroup (CI width: 4.4 dBA) compared to females (CI width: 2.1 dBA) reflects the smaller male sample (n = 49) and warrants cautious interpretation. Nevertheless, this pattern is consistent with previous studies showing that males are generally at higher risk of noise exposure.^15,24,36^ This pattern may reflect greater male participation in high-risk noise activities such as using power tools, riding motorcycles, and gaming.^37^

No meaningful difference in ANE was observed between participants with or without a medical background, with a median ANE of 70.9 dBA (95% CI = 69.7, 71.9) and 71.5 dBA (95% CI = 69.0, 73.3), respectively. The nonoverlapping CIs and small absolute difference of 0.6 dBA suggest the estimates are comparable across both groups. This study hypothesized a difference on the basis that individuals with a medical background may possess greater knowledge about the risks of NIHL. However, this awareness did not translate into reduced noise exposure or improved protective behaviors. This finding aligns with research indicating that behavioral change does not rely solely on health knowledge,^38^ particularly in the Malaysian context, where safe listening practices are not deeply embedded in cultural norms and hearing health has not traditionally been emphasized in school curricula.^39^

The small sample size of the Malay subgroup (n = 25) resulted in a considerably wider CI (95% CI = 66.9, 77.5, CI width: 10.6 dBA) compared with the Chinese subgroup (n = 180; 95% CI = 69.7, 71.7, CI width: 2.0 dBA), limiting the precision and interpretability of this comparison. The substantial uncertainty in the Malay estimate precludes any meaningful conclusion regarding racial differences in ANE in this study. Nevertheless, the possibility of racial differences in noise exposure warrants consideration. Supporting this notion, Mukari et al^40^ reported that among older Malaysian adults, hearing loss was less likely in Chinese compared with Malay individuals (OR = 0.56; 95% CI = 0.32, 0.98), suggesting that racial differences in auditory health outcomes may exist within the Malaysian population. While this finding does not establish a direct causal link between race and noise exposure, differences in the prevalence of hearing loss across racial groups may partly reflect cumulative differences in lifetime noise exposure. This raises the possibility that Chinese and Malay individuals in Malaysia may differ in their patterns of recreational or environmental noise exposure, warranting further investigation into noise exposure as a contributing factor to the observed racial disparities in hearing health. Internationally, ethnic variations in noise exposure patterns have been reported, with some minority populations experiencing higher exposure levels than white populations,^41^ and a larger, more racially balanced sample in future studies would enable a more meaningful comparison of ANE across racial groups in the Malaysian context.

### Hearing protection use pattern

Our findings revealed alarmingly low hearing protection use across all noise exposure activities, with approximately 85% of participants reporting never using protection during motorized vehicle use, amplified events, and musical instrument playing. These results align with global trends showing poor hearing protection adoption among young adults, despite the widespread availability and proven effectiveness of these devices.^12,42^ The consistently low usage rates across diverse activities suggest that barriers to hearing protection use extend beyond simple availability or awareness issues. Possible explanations include perceived inconvenience, concerns about reduced sound quality or social interaction, lack of immediate perceived risk, and insufficient emphasis on hearing conservation in Malaysian health education curricula.^43–46^ The finding that fewer than 10% of participants consistently used hearing protection across all activities is particularly concerning, given that many of these individuals had ANE values approaching or exceeding the 79 dBA risk threshold. Interestingly, participants with medical backgrounds showed no significant difference in protective behavior adoption, suggesting that knowledge alone is insufficient to drive behavioral change. This highlights the need for targeted interventions that address practical barriers and cultural attitudes toward hearing protection, potentially including education about modern, comfortable hearing protection options and strategies to normalize their use in recreational settings. Future public health initiatives should focus on developing culturally appropriate hearing conservation programs that move beyond awareness-raising to actively promote protective behavior adoption among Malaysian young adults.

### Reliability of modified Noise Exposure Questionnaire

The test-retest reliability of the modified NEQ was assessed using the ICC (ICC3 = 0.60, 95% CI = 0.22, 0.82), indicating moderate reliability. To our knowledge, Han et al is the only other NEQ validation study to report ICC-based test-retest reliability, with a notably higher ICC of 0.91 over a 1-month retest interval. The higher reliability observed in Han et al^21^ may partly reflect the shorter retest interval used in that study, as a 1-month gap is less likely to capture genuine changes in noise exposure habits compared with the 3-month interval employed in the current study. Additionally, the wide CI observed in the current study (95% CI = 0.22, 0.82) reflects the smaller subsample available for reliability analysis (n = 20), resulting in considerable uncertainty around the ICC estimate. Han et al^21^ did not report a CI around their ICC estimate, limiting a direct comparison of precision between the two studies. Other NEQ studies have assessed intra-subject reliability using repeated items within the questionnaire, reporting Kappa values of 0.59 and 0.55 by Johnson et al^20^ and de Oliveira et al,^47^ respectively; however, as this approach measures internal consistency within a single administration rather than stability across time, direct comparison with ICC-based test-retest reliability is limited.

### Limitations

This study has several limitations that should be acknowledged. First, we employed convenient and snowball sampling methods, which may limit the representativeness of the sample. Consequently, the findings may not be fully generalizable to the broader population of young adults in Malaysia. Future studies are encouraged to adopt stratified sampling based on relevant demographic characteristics, such as occupational background, to ensure more diverse participant representation. This approach would help achieve a more balanced sample and enhance the generalizability of the findings to the wider Malaysian young adult population.

The second limitation relates to the reliance on a self-report questionnaire to estimate ANE. Self-report measures are susceptible to recall and response bias, and genuine changes in participants’ noise exposure habits due to lifestyle, academic, or occupational shifts over the course of a year may introduce within-subject variability. Although the ICC estimate was moderate, the wide CI observed in this study reflects considerable uncertainty in the reliability estimate, attributable to the limited subsample (n = 20). The true reliability of the instrument could possibly range from poor to good, and a larger reliability subsample would be needed to obtain a more precise estimate. Further refinements of the ANE instrument should consider identifying and modifying items particularly susceptible to short-term variability. Validation against objective noise exposure measurements in a subset of participants would also strengthen the criterion validity of the instrument while retaining its practical advantages of low cost and accessibility.

Additionally, residential noise was not included as a separate activity category in the ANE calculation, as the high variability of residential noise levels, influenced by factors such as time of day, household composition, and neighborhood characteristics, makes standardization across participants difficult to achieve. This represents a gap in the current exposure estimate, as residential noise may contribute meaningfully to cumulative noise exposure, particularly among individuals living in urban or high-density environments. Future iterations of the ANE instrument could explore the feasibility of incorporating a standardized residential noise item to improve the comprehensiveness of the exposure estimate.

## Conclusions

This study has modified and validated the NEQ, incorporating culturally appropriate examples to assess ANE and hearing protection use patterns among Malaysian young adults. The findings revealed that 16.5% of participants exceeded the NIOSH-recommended limit of 79.0 dBA, indicating a significant risk for NIHL. Males demonstrated significantly higher noise exposure than females, while race and medical background showed no significant differences in ANE values.

Alarmingly, hearing protection use was consistently low across all activities, with 85% of participants reporting never using protection during high-risk activities. This finding is particularly concerning given that many participants approached or exceeded safe exposure thresholds.

These results underscore the urgent need for comprehensive hearing conservation programs targeting Malaysian young adults. Public health initiatives should focus on promoting safer listening practices and addressing barriers to hearing protection adoption, moving beyond simple awareness campaigns to culturally appropriate behavioral interventions. Future research should include more diverse populations and incorporate diagnostic hearing assessments to validate these self-report findings and guide evidence-based prevention strategies.

## ACKNOWLEDGEMENTS

We would like to express our gratitude to Prof. Tiffany A. Johnson for the permission to use the NEQ. AI assistance was used for manuscript editing and proofreading.

## Conflicts of interest statement

The authors declare that they have no conflicts of interest with regard to the content of this report.

## Supplementary Material


