# Supplementary material for: Annual noise exposure among Malaysian young adults
Source: Environ Epidemiol. 2026 Aug 10;10(4):e511. doi: 10.1097/EE9.0000000000000511 (PMC13461042; doi:10.1097/EE9.0000000000000511)
Supplement: Supplementary file 1 [file ee9-10-e511-s001.pdf]

Appendix 1

Instructions:<br><br>Please answer the following questions about yourself, hearing, and any noise you may have been around during the past year. Write an answer in the blank [________] or check [/] the best answer to each question.					
**Screening questions**					
1.	Are you clinically diagnosed with hearing loss?				
	☐ Yes	☐ No			
2.	Do you currently have ear diseases?				
	☐ Yes	☐ No			
3.	Are you aged within 19 to 39 year old?				
	☐ Yes	☐ No			
**Demographics**					
1.	Age (years)<br><br>*Eg: 22*				
	_____				
2.	Gender				
	☐ Male	☐ Female			
3.	Race				
	☐ Malay	☐ Chinese	☐ Indian	☐ Other:<br><br>_____	
4.	What is your highest level of education?				
	☐ SPM/ O-Level	☐ Pre-university/ A-	☐ Undergraduate	☐ Postgraduate	☐ Other: _____

		Level/<br>Diploma	(Master's /<br>Postgraduate)
5.	Are you currently studying or have you ever studied in the medical- or health-related field?		
	☐ Yes	☐ No	
6.	Are you currently working or have you ever worked in the medical- or health-related field?		
	☐ Yes	☐ No	
**General and Hearing Health**			
1.	Do you feel you have a hearing loss?		
	☐ Yes	☐ Maybe	☐ No
2.	Do you have trouble hearing or understanding conversations in noisy environments?		
	☐ Yes	☐ Maybe	☐ No
3.	Do you find it hard to concentrate in noise or loud environments?		
	☐ Yes	☐ Maybe	☐ No
4.	Do you find it difficult to hear and understand a speaker's announcement in noisy environments (eg: shopping mall, airports, train stations, etc)?		
	☐ Yes	☐ Maybe	☐ No
5.	Do you experience ringing, buzzing or hissing sounds (tinnitus) in your ears or in your head?		
	☐ Yes	☐ Maybe	☐ No<br>*(skip to #6)*
5a.	Does the ringing, buzzing or hissing sounds (tinnitus) last for at least five minutes each episode and occur at least twice a week?		

	☐ Yes ☐ No
6.	Are you more sensitive to everyday sounds compared to most people?
	☐ Yes ☐ Maybe ☐ No
7.	Do sounds ever seem distorted or unnatural to you?
	☐ Yes ☐ Maybe ☐ No
8.	Do you currently smoke any tobacco products or vape?<br>*Eg: cigarettes*
	☐ Yes ☐ No<br>*(skip to #9)*
8a.	If yes, please specify the type(s) of tobacco product and your average daily consumption:<br>*Eg: (i) Cigarette: number of pieces per day*<br>*(ii) Vape: number of sessions per day or milliliters of e-liquid used*<br>*(iii) Other:*
	________
9.	Have you ever had, or currently have any severe infections?<br>*Eg of severe infections: Rubella, sepsis, cytomegalovirus, meningitis, liver failure, tuberculosis, MRSA infection*
	☐ Yes ☐ No<br>*(skip to next section)*
9a.	If yes, please specify the infection(s).<br>*Eg of severe infections: Rubella, sepsis, cytomegalovirus, meningitis, liver failure, tuberculosis, MRSA infection*
	________

9b.	Did you take/ consume strong antibiotics for any of these infections as stated above?<br>*Eg of strong antibiotics: gentamicin, neomycin, paromycin, apramycin etc*
	☐ Yes ☐ Not sure ☐ No
**Noise Exposure Questionnaire (NEQ)**	
1.	For the past 12 months, did you work in a noisy paid job? Noisy job means sounds so loud that you had to shout or speak in a raised voice to be heard at arm's length.<br>*Eg: kitchen restaurant, workshop, factory, carwash, construction area, or other indoor or outdoor job working around loud equipment or machinery*
	☐ Yes ☐ No *(skip to #2)*
1a.	If you worked a noisy job, please estimate the number of weeks you worked in a year. There are 52 weeks in a year.
	________
1b.	If you worked a noisy job, did you wear earplugs or earmuffs at work?
	☐ Never ☐ Sometimes ☐ Always
2.	For the past 12 months, outside of a paid job, how often did you use power tools?<br>*Power tools mean the tools that produce very high sound levels. Eg: chainsaws, drills, nail*<br>*guns, compressors, and other shop tools.*
	☐ Never *(skip to #3)* ☐ Every few months ☐ Monthly ☐ Weekly ☐ Daily
2a.	If you used power tools, on average, how many hours did each time/session last?
	☐ 8 hours or more ☐ 4 hours up to 8 hours ☐ 1 hour up to 4 hours ☐ Less than 1 hour

2b.	If you used power tools, how often did you wear earplugs or earmuffs during this activity?				
	☐ Never	☐ Sometimes	☐ Always		
3.	For the past 12 months, outside of a paid job, how often did you drive heavy equipment or use loud machinery?<br>*Eg: tractors, trucks, lawn mowers, leaf blowers.*				
	☐ Never<br>*(skip to #4)*	☐ Every few months	☐ Monthly	☐ Weekly	☐ Daily
3a.	If you used loud machinery, on average, how many hours did each time/session last?				
	☐ 8 hours or more	☐ 4 hours up to 8 hours	☐ 1 hour up to 4 hours	☐ Less than 1 hour	
3b.	If you used machinery, how often did you wear earplugs or earmuffs during this activity?				
	☐ Never	☐ Sometimes	☐ Always		
4.	For the past 12 months, how often did you ride motorized vehicles such as motorcycles or four-wheelers/ all-terrain vehicles (ATVs)?				
	☐ Never<br>*(skip to #5)*	☐ Every few months	☐ Monthly	☐ Weekly	☐ Daily
4a.	If you rode motorized vehicles, on average, how many hours did each time/session last?				
	☐ 8 hours or more	☐ 4 hours up to 8 hours	☐ 1 hour up to 4 hours	☐ Less than 1 hour	
4b.	If you rode motorized vehicles, how often did you wear earplugs or earmuffs during this activity?				
	☐ Never	☐ Sometimes	☐ Always		

5.	For the past 12 months, how often do you take public transportation such as monorail, LRT, bus, subway or ferry?
	☐ Never *(skip to #6)* ☐ Every few months ☐ Monthly ☐ Weekly ☐ Daily
5a.	If you took the public transportation, on average, how many hours did each time/session last?
	☐ 8 hours or more ☐ 4 hours up to 8 hours ☐ 1 hour up to 4 hours ☐ Less than 1 hour
5b.	If you took the public transportation, how often did you wear earplugs or earmuffs during this activity?
	☐ Never ☐ Sometimes ☐ Always
6.	For the past 12 months, how often did you attend events with amplified public announcements or music systems?<br>*Eg: commercial/high school sporting events, music concerts, dances, karaoke centres,*<br>*clubs or car/truck races.*
	☐ Never *(skip to #7)* ☐ Every few months ☐ Monthly ☐ Weekly ☐ Daily
6a.	If you attended these events, on average, how many hours did each time/session last?
	☐ 8 hours or more ☐ 4 hours up to 8 hours ☐ 1 hour up to 4 hours ☐ Less than 1 hour
6b.	If you attended these events, how often did you wear earplugs or earmuffs during this activity?
	☐ Never ☐ Sometimes ☐ Always

7.	For the past 12 months, how often did you play a musical instrument?				
	☐ Never<br>*(skip to #8)*	☐ Every few<br>months	☐ Monthly	☐ Weekly	☐ Daily
7a.	What is/are the musical instrument that you played?				
	________				
7b.	If you played a musical instrument, on average, how many hours did each time/session last?				
	☐ 8 hours or<br>more	☐ 4 hours up<br>to 8 hours	☐ 1 hour up to<br>4 hours	☐ Less than 1<br>hour	
7c.	If you played a musical instrument, how often did you wear earplugs or earmuffs while playing?				
	☐ Never	☐ Sometimes	☐ Always		
8.	For the past 12 months, how often did you listen to music, radio programs etc. by using personal headsets or earphones?				
	☐ Never<br>*(skip to #9)*	☐ Every few<br>months	☐ Monthly	☐ Weekly	☐ Daily
8a.	If you listened through personal headsets or earphones, on average, how many hours did each time/session last?				
	☐ 8 hours or<br>more	☐ 4 hours up<br>to 8 hours	☐ 1 hour up to<br>4 hours	☐ Less than 1<br>hour	
9.	For the past 12 months, aside from music concerts or using headsets,<br>how often did you listen to music or radio programs through audio speakers,<br>such as in a car or at home?				
	☐ Never<br>*(skip to #10)*	☐ Every few<br>months	☐ Monthly	☐ Weekly	☐ Daily

9a.	If you listened via speakers, on average, how many hours did each time/session last?				
	☐ 8 hours or more	☐ 4 hours up to 8 hours	☐ 1 hour up to 4 hours	☐ Less than 1 hour	
10.	For the past 12 months, how often did you play games with loud background sounds using speakers, personal headsets or earphones?<br>*Eg: PUBG, Arena of Valor, Mobile Legend, RPG, race, shooting, strategy games etc.*				
	☐ Never	☐ Every few months	☐ Monthly	☐ Weekly	☐ Daily
10a.	If you played games, on average, how many hours did each time/session last?				
	☐ 8 hours or more	☐ 4 hours up to 8 hours	☐ 1 hour up to 4 hours	☐ Less than 1 hour	
